# Meeting Indigenous youth where they are at: knowing and doing with 2SLGBTTQQIA and gender non-conforming Indigenous youth: a qualitative case study

**DOI:** 10.1186/s12889-020-09863-3

**Published:** 2020-12-07

**Authors:** Billie-Jo Hardy, Alexa Lesperance, Iehente Foote, Native Youth Sexual Health Network (NYSHN), Michelle Firestone, Janet Smylie

**Affiliations:** 1 Well Living House, MAP - Centre for Urban Health Solutions, St. Michael’s Hospital, 209 Victoria St., 3rd Floor, Toronto, ON M5C 1N8 Canada; 2 Native Youth Sexual Health Network, 2345 Yonge St., PO Box 26069 Broadway, Toronto, ON M4P 0A8 Canada; 3 Dalla Lana School of Public Health, University of Toronto, 155 College St Room 500, Toronto, ON M5T 3M7 Canada; 4 Li Ka Shing Knowledge Institute, 209 Victoria St., 3rd Floor, Toronto, ON M5C 1N8 Canada; 5 Department of Family and Community Medicine and St. Michael’s Hospital Academic Family Health Team, 61 Queen St. East, Toronto, ON M5C 2T2 Canada

**Keywords:** Indigenous youth, Public health, Public health implementation research, Implementation science, Implementation research, Indigenous, Indigenous health

## Abstract

**Background:**

Research carried out in partnership with Indigenous youth at The Native Youth Sexual Health Network (NYSHN) demonstrates that Indigenous youth can (and do) develop and implement public health interventions amongst their peers and within their communities, when supported by non-youth allies and mentors.

**Methods:**

Together, NYSHN and Well Living House researchers co-designed a qualitative case study to demonstrate and document how Indigenous youth can and do practice their own form of public health implementation research (PHIR) in the realm of mental health promotion for 2SLGBTTQQIA and Gender Non-Conforming Indigenous youth. Academic and Indigenous youth researchers were: participant observers; conducted a focus group; and designed and implemented an online survey with Indigenous youth project participants. Governance, intellectual property, financial terms and respective academic and NYSHN roles and responsibilities were negotiated using a customized community research agreement. The data were thematically analyzed using a critical decolonizing lens that recognizes the historic and ongoing marginalization of Indigenous peoples while also highlighting the unique and diverse strengths of Indigenous communities’ knowledge and practice in maintaining their health and wellbeing.

**Results:**

Analysis revealed how colonialism and intergenerational trauma have impacted Indigenous youth identity and the value of self-determination as it relates to their identity, their relationships, health and wellbeing. We also learned how knowing and doing about and for Indigenous youth needs to be youth determined – ‘nothing about us, without us’ -- yet also supported by allies. Finally, our analysis shares some promising practices in knowing and doing for and with Indigenous youth.

**Conclusions:**

This study provides a reminder of the need to centre Indigenous youth throughout PHIR in order to realize sustainable benefit from research, services and programming. It emphasizes the need to recognize Indigenous youth as leaders and partners in these initiatives, support their efforts to self-determine, compensate them as partners, and prioritize Indigenous youth-determined frameworks and accountability mechanisms.

**Supplementary Information:**

The online version contains supplementary material available at 10.1186/s12889-020-09863-3.

## Background

Research, carried out in partnership with Indigenous youth at The Native Youth Sexual Health Network (NYSHN), demonstrates that Indigenous youth can (and do) develop and implement public health interventions amongst their peers and within their communities, when supported by non-youth allies and mentors [1–3]. Key factors for success, demonstrated through past projects include: support for youth leadership; prioritization of cultural relevance and safety; and a focus on strength-based methods [1–3]. Despite this evidence, the voices, contexts and expertise of Indigenous youth continue to be underrepresented in public health research, programs and services. The result of which is research harms [4] and suboptimal public health programming and services for Indigenous youth [5]. Against this background, one might argue that the persistent health inequities experienced by Indigenous youth, (e.g. higher rates of suicide and mental health conditions [6–8]) are, at least in part, a manifestation of the failure to include Indigenous youth in the development, implementation and scale up of interventions directed at them.

Investments in implementation science (IS) and more specifically, public health implementation research (PHIR), provide a possible avenue to remedy these challenges, in that the method “seeks to understand work within real world conditions” [9]. As such, the context surrounding an intervention and the active involvement of end-users are of central importance; reconciling interventions with local realities. Canadian Institutes of Health Research (CIHR) funding programs promote IS in Indigenous health research [10]. Although these frameworks promote a shift from more descriptive approaches to actively addressing Indigenous health inequities, the theories and methods of IS and IR in public health have been developed outside of Indigenous contexts. As a result, they must also be systematically reconciled with existing Indigenous knowledge systems and assumptions.

In this paper, we present a qualitative case study, that applies an Indigenous case study method [11, 12] and partners with NYSHN team members who co-led a youth-determined Indigenous IS project entitled, ‘Youth-governed approaches to mental health promotion and suicide prevention for Two Spirit, lesbian, gay, bisexual, transgender, queer, questioning, intersex and allies (2SLGBTTQQIA) and Gender Non-Conforming Indigenous youth’. The intentional placement of Two Spirit (2S) at the front of the acronym serves as an act of decolonization, to reclaim and prioritize chosen Indigenous identity in an otherwise white-washed movement that takes place on stolen Indigenous land. 2S is used by some Indigenous people to express our diverse cultural and ceremonial roles and responsibilities in relation to our traditional understandings of gender and sexuality. These understandings will look different from nation-to-nation and each person’s teachings will inform their experience and identity. Some Indigenous people who identify as Lesbian, Gay, Bisexual, Transgender, Queer, and/or Questioning might identify as 2S as well. Others might not. Being Two-Spirit does not literally mean ‘having two spirits,’ but some people do identify as having a spiritual balance of both feminine and masculine energies. Two-Spirit is used by and for Indigenous people as a way to relate to ourselves, our communities, and our spirits outside of a western colonial context. Though colonialism attempted to erase the roles of 2S community members, this term serves as a starting place to begin the work of healing, recovering, and reclaiming our spaces in the circle.

The above-mentioned CIHR funded PHIR research project [13] was purposefully designed in partnership with NYSHN to demonstrate and document how Indigenous youth can and do practice their own form of PHIR in the realm of mental health promotion for 2SLGBTTQQIA and Gender Non-Conforming Indigenous youth. To achieve this, academic and Indigenous youth researchers acted as participant observers; conducted a focus group; and designed and implemented an online survey with Indigenous youth project participants. Through centering Indigenous youth-determined experiences, we build on learnings from previous research [1–3] and lend insight towards promising practices for conducting PHIR with- and for- Indigenous youth. We also identify the ways health and wellness behaviours can be promoted through services and barriers to those behaviours.

## Methods

### Theoretical framework and core research assumptions

**Fig. 1:**
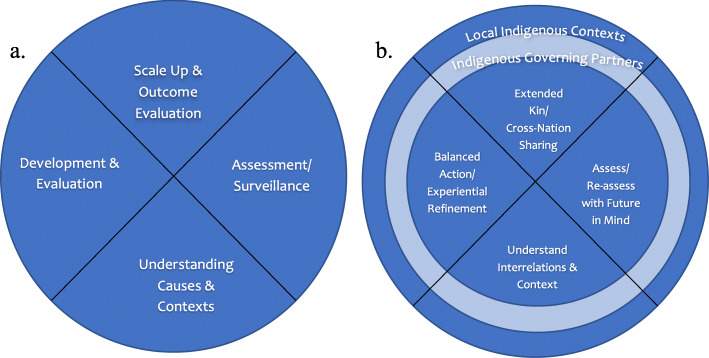
**a** Core Public Health Implementation Research Steps and **b** Indigenous Implementation Science Model

The theoretical framework underlying this work builds on an Indigenous reframing of the existing core steps of PHIR advanced by the senior author as the platform for a larger research program in which this case study was nested (Fig. 1a and b). Our starting assumption is that Indigenous communities have already been practicing their own unique and diverse forms of Indigenous IR. Sophisticated and systematic processes for planning and iteratively evaluating collective actions to enhance wellbeing are embedded in historic and contemporary Indigenous epistemologies and societies. Historically, and currently, there are rich traditions of sharing practices and technologies across distinct Indigenous communities and nations. Community visiting; inter-nation gatherings; and now information technologies are ways in which diverse approaches and practices are shared. Before an idea or innovation is locally implemented, in our experience, there is commonly a careful analysis of potential local utility that builds on intrinsic understandings of local ecologic, cultural and social systems. Our baseline Indigenous IR model (Fig. 1b) asserts an Indigenous epistemological framework in which the key steps of PHIR are re-formulated and articulated to reflect the senior author’s understanding of key relevant tenets and assumptions. In addition to our starting premise described above, these include:

- Indigenous community leadership and investment are critical mechanisms underlying successful health interventions for Indigenous infants, children, youth and their families [14, 15].
- Successful Indigenous health interventions are tailored to- and build on- local Indigenous values, skills, knowledge, and beliefs [11, 14].
- Relationality (the interconnection between all things) reciprocity (mutual exchange) and balance at the individual, interpersonal, collective, and socio-ecological levels are key concepts essential to successful Indigenous health services, research processes and capacity strengthening [16].
- A multi-generational perspective that is informed by one’s ancestors and takes into account the health of future generations is central to day to day analysis and decision making [17].
- Wisdom is achieved through the collective and iterative sharing, uncovering, and interweaving of iterative empirical observation, lived experience, dreams and linked metaphysical manifestations [18, 19].

This framework challenges historic and ongoing marginalization of Indigenous peoples in the planning, implementation and assessment of Indigenous health research, services, and policies and the external imposition of non-Indigenous models premised on non-Indigenous epistemologies and knowledge assumptions. We are motivated in our desire to interrupt the systemic application of non-Indigenous approaches since this is known to be ineffective [14, 20, 21]. This specific youth-governed Indigenous mental health promotion project by and for 2SLGBTTQQIA and Gender Non-Conforming Indigenous youth and linked case study aims to advance, refine, and contextualize the more general baseline framework to the Indigenous IR approach used by NYSHN to promote mental health and wellbeing among 2SLGBTTQQIA and Gender Non-Conforming Indigenous youth.

### Research approach

The research team was comprised of Indigenous and allied Indigenous health scholars and NYSHN Indigenous youth researchers. Working together we applied an Indigenous research partnership approach that has been iteratively developed by the academic team at Well Living House (WLH) over the past decade [11, 22, 23], tailoring it for this specific project and partnership. As part of this approach, the Indigenous academic and NYSHN Indigenous youth research leads invested in developing, strengthening, and sustaining our interpersonal relationships by applying Indigenous kinship terms and protocols. Governance, intellectual property, financial terms and respective academic and NYSHN roles and responsibilities were then negotiated using a customized community research agreement (See Additional files 1 and 2) that acknowledged the cultures, languages, knowledge, values, and rights to self-determination of NYSHN and supported implementation of Indigenous collective, and self-determined, data management and governance. NYSHN was identified as the custodian of all data gathered from NYSHN membership in the project.

### Research partners

The Native Youth Sexual Health Network (NYSHN) is a grassroots organization, formed in 2010, by and for Indigenous youth, that works across issues of sexual and reproductive health, rights and justice throughout the United States and Canada. It is collectively led by and for Indigenous youth who are 30 years of age and under. In addition to its own membership, NYSHN is advised by 3 Youth Councils: National Indigenous Young Women’s Council, National Indigenous Youth Council on HIV/AIDS and National Native American Council on HIV/AIDS. The NYSHN model holds space for all members of NYSHN, youth councils, partner organizations as well as aunties, uncles and mentors. The model fosters sustainability because it has built-in core support for core youth members and a built-in model for working with those people who have history and memory with NYSHN.

Well Living House (WLH) is an action research centre for Indigenous infants, children, and their families’ health and well-being. Our focus is on gathering, using, sharing, and protecting Indigenous health and well-being knowledge and practices. We draw on both Indigenous and public health knowledge to inform cutting edge scholarship and best practices. At the heart is an aspiration to be a place where Indigenous people can gather, understand, and share what it means to be a healthy child, family, and community – building a “Well Living House”.

### Indigenous community and research ethics board approval

The study protocol was vetted and approved by NYSHN in keeping with the agreed-upon roles and responsibilities outlined in SMH-NYSHN Research, Data Sharing, and Publication Agreements (See Additional files 1 and 2). The protocols were also approved by the Unity Health Toronto Research Ethics Board (study nos.:15–033 and 15–175).

### Study design

NYSHN Indigenous youth researchers and WLH researchers co-designed the study, applying a previously demonstrated qualitative Indigenous case study design [11, 12] to provide an in depth, naturalistic understanding of how Indigenous youth involved with NYSHN are working together to advance the health and wellbeing of Indigenous youth. Specifically, we sought to truthfully articulate the health promoting strategies and actions that Indigenous youth were applying among themselves along with key contextual barriers and facilitators to their work. Working closely with NYSHN Indigenous youth researchers we aspired to draw on conceptual frameworks and language that Indigenous youth themselves use to make sense of themselves and their worlds.

In keeping with the need to respect the distinct histories, languages, cultural practices and life stages of diverse Indigenous communities, the unit of analysis for our Indigenous case study methodology is the community or organization (in this case NYSHN) and our methods are primarily intrinsic according to case study methods more generally [12, 24]. As mentioned earlier, this specific case study is one of a series of case studies comprising a larger research program aimed at advancing the theory and practice of Indigenous implementation research more generally, so there is also an instrumental component.

Specific objectives for this NYSHN PHIR case study are:

- documentation of Indigenous youth-determined conceptual models and practical strategies for research partnerships and platforms;
- identification of barriers to- and facilitators- of health and wellness promoting behaviours, including the role of underlying determinants of health including racism, family and community relationship and health literacy, and existing systems of health knowledge and practice;
- supporting the assessment of promising practices; and,
- sharing of best practices across Indigenous communities.

### Data collection

In keeping with qualitative case study methods [12, 24], the WLH and NYSHN research teams collected and triangulated data from multiple sources over a two-year time-span (2015–2017), including: a focus group, an online survey, NYSHN draft governance policy document, the NYSHN website, as well as research team reflections and observations. All data collection tools (focus group questionnaire and on-line survey were co-designed by the WLH and NYSHN research teams in order to reflect on the underlying values of NYSHN and WLH (i.e. focus on strengths as opposed to deficits).

The focus group was held in 2015 at the Youth-Governed Approaches to Mental Health Promotion and Suicide Prevention for 2SLGBTTQQIA and Gender Non-Conforming Indigenous Youth Gathering in Toronto, Ontario. It was co-facilitated by two Indigenous community members who were chosen based on their experience as facilitators and their ability to prioritize the comfort of the youth participants and provide peer support where requested. The focus group sample included seven (*n* = 7) Indigenous self-identified 2SLGBTTQQIA youth (i.e. NYSHN youth leaders and members) and lasted 1 h and 20 min. The focus group was audio-recorded, transcribed verbatim and subsequently validated by NYSHN.

In lieu of an additional planned focus group, an online survey was administered in 2016 by the NYSHN co-leads in order to accommodate participant schedules and capacities as well as varying comfort levels with research across the membership. Five NYSHN team members who were, at the time, actively involved in NYSHN organizational and research activities were invited to complete the anonymous, online survey. All five (*n* = 5) invited members completed the survey, many of whom identify as 2S. Participants were asked to identify and reflect on barriers and facilitators to health and wellness. The survey took approximately 20–30 min to complete and was validated by NYSHN.

Key paper and online documents were collected to enrich the focus group and survey data. These included a draft NYSHN policy document that describes the internal governance, organization and priorities of NYSHN as well as the NYSHN website. The NYSHN website [25] outlines NYSHN’s mandate and approach and provides access to materials (i.e. published research) related to research and service-based activities in which NYSHN leads and/or partners on. Researcher reflections and observations were generated throughout the study to record the behaviours, activities, events and observations of the case study. All participants were 16 years of age or older and provided written or verbal informed consent prior to participating in the focus group and/or online survey and each received an honorarium to acknowledge their contributions.

### Data analysis and writing

The data were analyzed using thematic analysis [26] and a critical decolonizing lens that recognizes the historic and ongoing marginalization of Indigenous peoples while also highlighting the unique and diverse strengths of Indigenous communities’ knowledge and practice in maintaining their health and wellbeing [11, 22, 23]. Following completion of data collection activities, a WLH and NYSHN researcher were selected by the larger research team to co-analyze the transcripts, online survey responses and draft NYSHN policy document as well as write up the results. The researchers met twice to review and discuss the case study setting and data and set a plan for analysis. Afterwards, the WLH research partner coded the transcripts, surveys and draft NYSHN policy document, beginning with open coding, in which the focus group transcript, completed surveys and NYSHN governance document were manually coded. The WLH and NYSHN researchers then met on several occasions to discuss the codes, reconcile them and group them into categories and then into themes. Case study notes of researcher reflections and observations as well as the NYSHN website were used to triangulate the data and final themes were validated through the convergence of different perspectives resulting in theoretical saturation. A draft outline of the preliminary themes and results were iterated through two separate interactive sessions: one in November 2016, with NYSHN members at a NYSHN gathering in Toronto, ON and another in September 2017, with Forum for Indigenous Implementation Research and Evaluation (FIIRE) Network members at a FIIRE gathering in Whitehorse, YK.

## Results

We present the results here as 4 overarching and interconnected themes: Indigenous youth identity; health and wellbeing of Indigenous youth; knowing and praxis about and for Indigenous youth needs to be youth determined – ‘nothing about us, without us’; and, promising practices in knowing and doing for and with Indigenous youth.

### Indigenous youth identity

In the focus group session, Indigenous youth participants explained that authentically defining youth is challenging and can leave them feeling isolated or excluded. One participant, for instance, pointed to how age was a poor descriptor.

‘I also think someone’s going to have to back it up by like what do we mean by youth because I don’t know that everyone is always on the same page as that. I’m increasingly not sure that that is determined by age.’ [FG:6]

Others spoke about the experience of transitioning into and out of ‘youth’ and the challenges this can present to maintaining their networks. Some shared for example, how they are struggling to facilitate aging out of ‘elder’ youth from NYSHN and identified the need for ‘pastoral’ and ‘old’ youth in order to maintain their chosen networks. NYSHN aims to address these challenges and is developing mechanisms to accommodate existing members while also welcoming new members. For instance, NYSHN members are working to incorporate Indigenous ceremonies (i.e. Rites of Passage and Coming of Age) into their practice to mark a transition into and out of youth.

On the whole, participants considered youth to be a dynamic and transitional time. In particular, they described Indigenous youth as: resistant; survivors; inclusive; heterogeneous; and, intersectional.

Participants in the focus group discussed the effects of intergenerational trauma on identity and how Indigenous youth are sometimes excluded from the narratives of colonialism. They shared their unique experiences with colonialism and how it acts to disconnect Indigenous youth from family and community, informing their identities.

‘[ … ] the violence from these various systems happens from birth. Bad birth doctors’ separate newborns from their parents, then Child and Family Services steals them, and then educational institutes separate Indigenous youth from their parents, communities, nations.’ [S:2]

Participants explained that as a result, Indigenous youth have been disconnected, oftentimes right at birth. As a result, we were told that meaningful and purposeful partnerships with Indigenous youth are integral to the development and implementation of sustainable and effective research, service and programs directed towards Indigenous youth. Partnering with youth, as advised by NYSHN and as represented in their governance, goes towards re-establishing intergenerational connections, in part, through inclusion, support and respectful relations.

### Health and wellbeing of Indigenous youth

#### Health and wellness for Indigenous youth is poorly defined

In general, the focus group, survey and documents all indicated that Indigenous youth health and wellbeing is complex, contradictory, dynamic, and subject to situational and environmental influences.

Participants cautioned that Indigenous youths’ health and wellness has been poorly defined by the public health research community who disproportionately rely on research-based metrics, like height, weight, disease, etc. As noted by one participant, ‘[ … ] even if all these things were met, I’m not sure that it [health] would be defined as good, since it is dependent on what is going on in someone’s individual life; rather than the boxes that they ‘check’ of basic health and wellness or what someone else defines is going on for them.’ [S:1] These measures, we were advised, are too restrictive, focusing primarily on the individual and failing to recognize how, for Indigenous youth, health and wellness is interrelated with their communities and relationships, i.e. kinship networks.

Participants in the focus group listed several aspects that, in their experience, influence Indigenous youth health and wellbeing, including: self-determination; harm reduction; intergenerational and peer support; validation; connection; and, relationships and cultural safety.

According to many of the participants, poorly informed research contributes to ineffective, paternalistic public policy and service provision for Indigenous youth in Canada. Participants were clear that for Indigenous youth, the conversations can only begin by recognizing that health and wellness must be self-determined. For example, they explained that given the opportunity, Indigenous youth are more likely to focus on harm reduction over treatment services. Harm reduction is defined by NYSHN as a way of life, “that we, as well as our ancestors, have been keeping our communities safe and reducing harms long before the word ‘harm reduction’ came into the English language. It’s about reducing the many harms in our lives, not limited to just substance use (i.e. colonialism, racism, homophobia/transphobia, criminalization, etc.) through the tools that work best for us, without stigma or judgement. We also don’t define what harm is for other people.” [http://www.nativeyouthsexualhealth.com/whatwebelievein.html]. Participants were quick to point out however, that in order to self-determine Indigenous youth still require support, skills and tools.

“ … self-determination doesn’t mean totally leaving people alone (unless they explicitly say so) but holding space for their voice to matter and be heard.” [S:1]

**Fig. 2:**
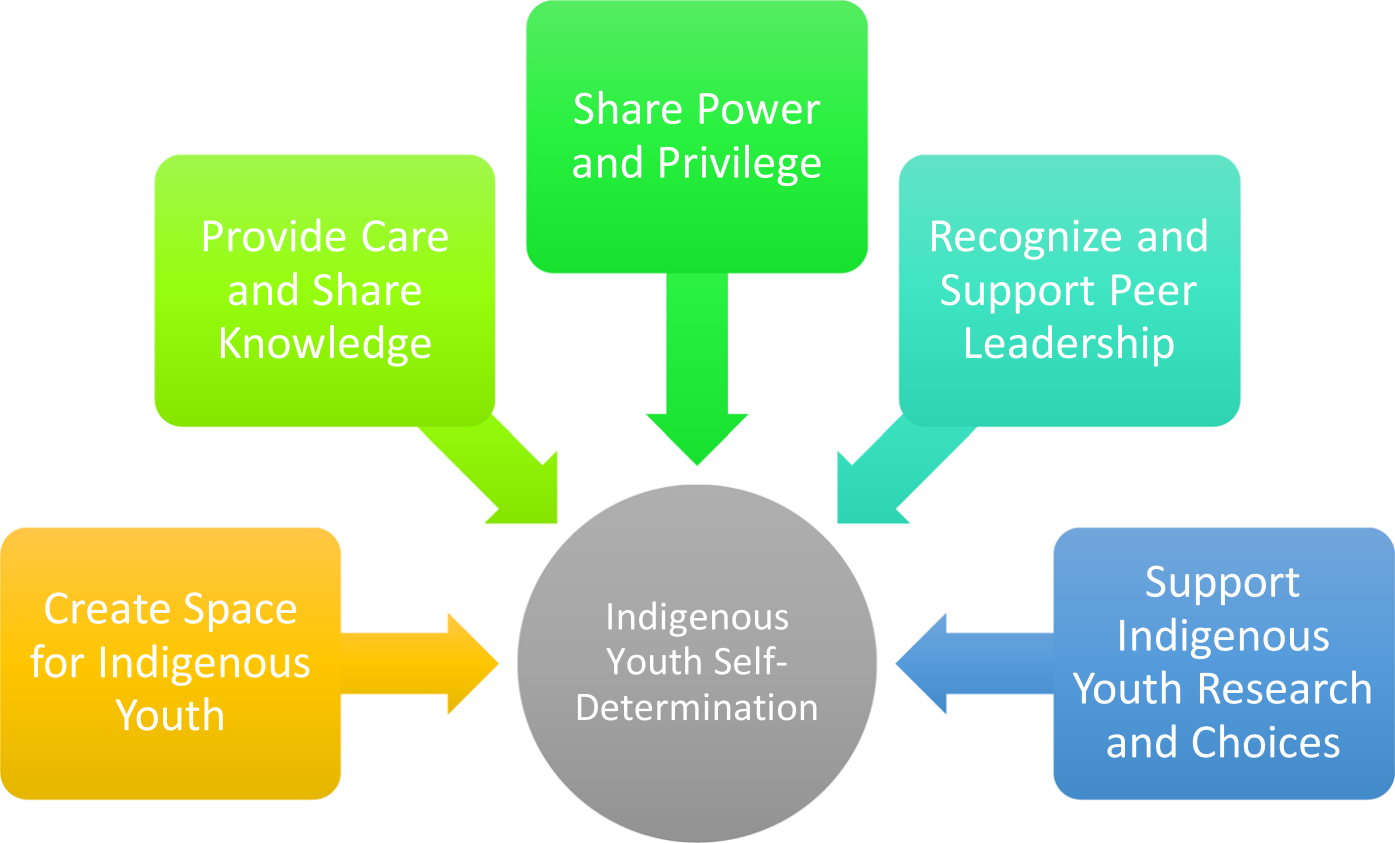
How to support and facilitate self-determination with Indigenous youth

Figure 2 provides some insight, as described by participants, both in the focus group and the online survey as well as in the NYSHN draft policies and website, as to how non-youth mentors, allies and communities can support and facilitate Indigenous youth to self-determine.

#### Indigenous youth know when they (or their peers) are unwell

Some participants struggled with defining how or whether they had ever experienced ‘good’ health and wellbeing. One participant described how ‘[ … ] Indigenous youth are in perpetual state of un-wellness.’ [S:3] This insight was shared by others and reflects how the persistence of colonialism and intergenerational trauma experienced by Indigenous youth in Canada has been normalized. Alternatively, one participant drew attention to hegemony, noting that who and how they define what qualifies as unhealthy for Indigenous youth should be approached with caution.

‘I’m not sure what good health and wellness looks and feels like. I can get through the day I’m supported, when I’ve slept, when I’ve drank water, ate some food, and tried my best to listen and interpret what my body is saying.’ [S:4]

‘[B] eing unhealthy or unwell is a completely natural response to the many violences constantly inflicted on our communities.’ [S:3]

Participants from the online survey identified changes in behavior and disconnecting from friends and family as examples of early signs that peers may be unwell. Participants also identified youth kinship networks as youth-mediated means for identifying when other youth are unwell and in need of an intervention.

#### Relationships are critical to Indigenous youths’ health and wellbeing

Almost all participants shared how diverse relationship networks were critical to their health and wellbeing. Participants also spoke of how their relationships are often self-determined, and include a mixture of some or all of the following: chosen family; biological family; ancestors; land; and, service providers. Indigenous youth well-being is impacted when these relationships are disrespected through research and are prescribed, mandated or disrupted through social and public health services. Examples include early separation from bio-families and disrespecting or failing to recognize sexual identities or chosen kin.

‘Disruption of family and community relationships, including and especially those of family of choice, ceremony and adoption, play huge roles in how young people see themselves and in particular their mental and spiritual health. Particularly for young people who have vocalized, in small or big ways, who family is to them or who their kinship network is they want to be with and are not allowed – either by law or by circumstances of lack of support.’ [S:1]

These disruptions can begin early for Indigenous youth, as described by one participant who said, ‘the disruption of having people taken away from you too early is so harmful, i.e. your big supports pass away too early or youth are relocated or taken away by Child and Family Services.’ [S:2].

The effect for some, as one participant pointed out, can be ‘traumatizing and sever folks from health supports.’ [S:5] Alternatively, however, the same participant went on to suggest that it can also be positive and liberating, in that disruption encourages self-determination. The participant noted, providing NYSHN as an example, that it might be ‘[ … ] a place where new families are made.’ [S:5] The ability of Indigenous youth to identify and access alternative relational supports is critical and depends mainly on the health and diversity of these networks.

For these reasons, participants maintained that, supporting Indigenous youth to build and sustain these networks and acknowledging their choices are crucial to respecting their chosen identities.

‘[I] t is just as important for young people to define what family and community is to them and who their peers are.’ [S:1]

‘[ … ] supporting young peoples to make decisions about who their community and family is contributes to good health and wellness.’ [S:4]

Participants noted that non-youth allies and mentors have a responsibility to ‘uphold these relationships and to protect them from all other colonial and systemic interferences that try to say they don’t matter as much.’ [S:1]

### Knowing and praxis about and for Indigenous youth needs to be youth determined – ‘nothing about us, without us’

The focus group, survey, and the NYSHN website and documents all promote that knowing and praxis in public health for and about Indigenous youth (i.e. IS and PHIR) must be youth-determined throughout the entire process. From priority setting to implementation and on through to health promotion and service delivery. Participants noted that, in their experience, many of the services and programs available to youth are, in fact, not youth-determined, and instead have the effect of further isolating Indigenous youth.

‘I often find myself following things back to see if they are actually youth-led. And unfortunately, more often than not, they’re not.’ [F:1]

Participants maintained that when knowing and praxis are instead Indigenous youth-determined, the results are diverse, inclusive, accessible, flexible, decolonizing, culturally safe and accountable to Indigenous youth. As an example, focus group and survey participants stated that priority setting should be led by- or undertaken in partnership with-, Indigenous youth. As put by one participant, ‘[ … ] having the agenda set by youth is, you know, key to everything.’ [F:3] Indigenous youth know what they need as well as the tools to get there and these are reflected in the NYSHN beliefs, principles and practice. However, as participants pointed out, priorities are mainly set by non-youth, resulting in an unequal division of power between those parties and Indigenous youth. Supporting youth-driven priorities requires the meaningful engagement of Indigenous youth in their communities, separate from the involvement of community leaders (i.e. chief and council).

Systemic barriers in research were also identified as creating challenges to youth leadership. For the most part, participants viewed funding opportunities as biased to favour academics as opposed to youth and their communities. Participants stressed that funding for research should instead be more readily available and accessible to Indigenous youth. They noted that most direct funding opportunities that do exist for Indigenous youth are minimal and capped. As a result, participants described feeling pressured into having to develop relationships with researchers to pursue projects. One participant pointed out how, ‘all of our access to CIHR in the last ten years has had to be with academic institutions and the network (NYSHN) still can’t apply [ … ] our youth still can’t apply to research projects on their own.’ [F:7] Participants pointed to how these are difficult relationships to negotiate, particularly if there is a disconnect between what Indigenous youth are hoping to achieve versus what a researcher might be interested in. As an organization, NYSHN has structured itself to unsettle this by, ‘… trying to reverse that flow and say okay, this is what we’ve seen the need for, this is what youth are asking for and now how do we get the resources to make that happen?” [F:1]

At the same time, focus group participants stressed that ‘[ … ] youth-led doesn’t mean that we put them on an island all by themselves like with no help or support, because I think that’s equally problematic.’ [F:6] Instead, participants pointed out that the role of adults and allies is to support and enable Indigenous youth leadership to facilitate them when setting their own priorities. As suggested by one participant, adults and allies need to ‘[ … ] challenge other adults to stop talking for, on behalf of, or making decisions for and on behalf of young folks.’ [F:2] The participant went on to say that, Indigenous youth need to be empowered to describe to their chosen allies (i.e. within the community, government or research institutions and service-oriented organizations) that ‘[ … ] this is the way I need your help.’ [F:2]

Participants recommended that adults create safe spaces where Indigenous youth can share their concerns and ideas with each other and with non-youth. As explained by one participant, ‘just providing that safe space for them to talk and listen to them [Indigenous youth]’. [F:4] They also suggested that adults be more culturally safe. In many cases, research with and about Indigenous youth has been, and continues to be, unsafe, as it fails to acknowledge colonization and its associated traumas. Rather, research frequently reinforces trauma through linking the experience of violence to the cause of violence or repeatedly researching the cause of suicides as opposed to solutions. Participants suggested that research can be more culturally safe when it acknowledges and accounts for peer support systems and inter-generational trauma that is the result of colonization. Peer programming, we were told, should form an integral part of any project or service because it acknowledges ‘[ … ] that’s just as much a part of the process of a project or program is that we show up for each other because oftentimes, nobody else is showing up for us.’ [F:7] Another critical suggestion made was to stop all research programs that are rooted in reinforcing trauma.

In Table 1 we summarize the practical suggestions shared by participants in both the focus groups and surveys for how health services and programs for Indigenous youth as well as health research for and with Indigenous youth can be improved upon.

**Table 1:** Practical suggestions to improve health services and research for and with

Health Programs and Services for Indigenous youth	Research for and with Indigenous Youth
Programs and services intended for Indigenous youth need to provide safe spaces for Indigenous youth expertise	Research with and about Indigenous youth needs to be culturally safe
Program and service priorities and agendas need to be informed by Indigenous youth expertise	Research with and about Indigenous youth should be inclusive andopen-ended
Invest financially in Indigenous youth and peerleadership for programs and services	Indigenous youth must be partners in research with and/or about them
There needs to be more visibility for what works for Indigenous youth	Research with and about Indigenous youth must be wellness-based
	Good research is sustainable in that it builds relationships with Indigenousyouth, sharing roles, responsibilities and opportunities as partners; empowersyouth; and, respects their interest in self-determination

### Promising practices in knowing and doing for and with Indigenous youth

There are ‘knowing and doing’ interventions that meaningfully engage and are viewed positively by Indigenous youth. These interventions are considered promising practices. One participant, for example, shared their experience with a food sovereignty and mental health garden project.

‘It was for Indigenous youth of colour where youth didn’t have to prove their identity’, it was peer led with a youth circle and an aunties and uncles circle. It was about addressing mental health by participating in organic gardening for remedies that helped feed families and also harvested medicines for mental health itself. Over and over again, I heard young people say they felt they were being heard, they felt safe, and they felt like what they were doing mattered – to their own health, and their communities health.’ [F:1]

Others pointed to the Sexy Health Carnival: ‘Sexy Health Carnival is peer-led, provides accessible information, resources and space to learn to have fun and engage with information that made most sense to you.’ [F:4] An example of research practices that centre Indigenous youth’s leadership and ideas, as well as mentoring to strengthen Indigenous youth’s capacity, the Sexy Health Carnival was initiated by NYSHN youth facilitators, working with their community. The carnival works to break the barriers of fear, stigma and shame relating to issues that Indigenous youth face in their communities. It has created safer practices content that makes learning information about issues that affect our bodies more accessible and most importantly more fun to learn about for inspiring youth, community members and Elders. The idea to broaden the scope of the carnival came from Indigenous youth – including the creation of boards and supports. Various booths range from topics such as suicide, harm reduction, consent, sexual violence prevention, STI’s, birth control and masturbation.’ [http://www.nativeyouthsexualhealth.com/sexyhealthcarnival.html] The carnival also features interactive games, prizes and safer sex supplies, as well as content and age-appropriate activities for younger children so that their parents, siblings or other care-takers can participate in the carnival. It has become one of NYSHN’s most popular outreach tools, with many requests each year from Indigenous communities for the carnival to visit their community. Indigenous youth leaders conducted evaluation of the program in 2014 with funding from Ontario HIV Treatment Network (OHTN) and received the OHTN Community-based Research Award. They have also received a CIHR award to expand implementation and evaluation across additional communities. Critical to this example is how the community goals differ from those of research. Here Indigenous youth and their communities initiate their own Sexy Health Carnival and mentor youth to be able to do this work in comparison to research goals, one of which includes addressing gaps in the literature that connect sexual health to wellbeing, culture, etc.

Table 2 is a summary of the features of promising practices that apply to IS and PHIR.

**Table 2:** Features of promising practices that apply to IS and PHIR for and with Indigenous youth

Meet youth where they are at	Be supportive of Indigenous youth, not judgmental. Knowing and doing should be focused on harm reduction with Indigenous youth
Culturally safe	There should be no stigma, shame and blame. Knowing and doing should enable learning.
Wellness–based and fun	Knowing and doing should be uplifting and fun – Indigenous youth are inspiring and lookingfor inspiration.
Accessible	Knowing and doing needs to be accessible. Efforts should be made to tailor language andcommunication tools in ways that speak to Indigenous youth (e.g. social media). Also,programs and services need to lower barriers – Indigenous youth should not need toidentify as at-risk to be eligible as it further victimizes them and as an approach it often failsto acknowledge its basis in colonialism.
Youth-determined	Empower youth and allow them to define what this means to them as it is both contextualand diverse.
Peer and Youth-led	The most promising and relevant knowing and doing is led by youth. At the very least, itshould be partnered with youth, where they have clear roles and responsibilities andopportunities for mentorship and leadership. These roles would apply to management andgovernance over funding and resources.
Resourced	Knowing and doing about, for and with Indigenous youth needs to be well resourced. Fundsneed to be allocated toward engaging and partnering with youth, compensating youth, andproviding opportunities for training, mentorship and employment. Youth should and need tobe involved in improving their well-being and these contributions should not be undervaluedor undermined by a lack of available resources.

## Discussion

Crafted with Indigenous youth, this case study documents the perspectives, experiences, and observations of Indigenous youth participating in implementation research. Our findings highlight the need to support youth leadership, prioritize cultural relevance and safety, and focus on strength-based methods in Indigenous youth PHIR. The findings complement previous work [1–3], offering key insights, promising practices, and future direction for research and program and services in public health.

Our discussion is focused on the following insights for IS and PHIR involving Indigenous youth:

- Indigenous youth should be supported to lead as equal partners in efforts to describe or define their own complex identities and experiences;
- Indigenous youth need to be empowered to self-determine;
- knowing (research) and doing (programs and services) with Indigenous youth should not be approached as discrete activities; and,
- IS and PHIR need to be accountable to Indigenous youth.

Throughout this study, participants raised concerns about the ways in which “youth” are defined. Youth and their experiences are often assigned, described and defined by adult-led organizations and agencies and not by youth. For example, the United Nations (UN) ‘for statistical purposes’ defines ‘youth’ as ‘those persons between the ages of 15 and 24 years.’ [27] These definitions have impact. Participants think there is a direct link between the reductionist ways by which they are defined (e.g. age) and their limited access to research opportunities, programming and services intended to meet and address their needs. Participants in this case study view these processes as paternalistic, exclusionary, and contradictory to their principle of Indigenous youth self-determining their identities. NYSHN, has had to innovate in order to be recognized as a legitimate youth organization by government agencies and funding institutions (e.g. by categorizing members who fall outside of the defined age group as ‘pastoral’, ‘elder’ and ‘old’ youth). In doing so, NYSHN has been able to maintain critical intergenerational relations, through their model, with members who are ‘in transition’ but are still providing continuity and mentorship to other Indigenous youth members – an Indigenous framework like that described by Anderson ([17]:168), “Within the human domain, the way in which community members connected across the generations was critical to the health and well-being of the present-day and future community. Relationships between elders and children were considered critical in terms of maintaining the life force and survival of the people and future community. Relationships between elders and children were considered critical in terms of maintaining the life force and survival of the people …” .

Similarly, participants were critical of the motivations driving existing research on the health and well-being of Indigenous youth, from which they have been systematically marginalized. In large part, due to the ongoing deficit-based categorization as vulnerable. Participants viewed much of the research as enriching the careers of others and being largely informed by reductionist, deficit-based metrics that fail to capture the complexities of Indigenous youth and their experiences. These views align with the call for research on public health interventions that respects cultural beliefs and practices [28] and recognizes that First Nations and Métis Indigenous youth ‘understand health in a holistic manner’ that is linked to culture and community [6]. They are also in line with recent scholarship [1–3, 29, 30] that promotes strength-based approaches in research with and about Indigenous youth. Danforth [27] stresses that “being Indigenous, or being a young person, is not a ‘risk factor’ by itself. In fact, being ourselves can be empowering … ’. Instead, Danforth argues, it is the influence of colonialisms, adultism, and racism as well as a lack of access to culturally safe space that puts the lives of Indigenous youth at risk.” [31] Finally, according to participants, these definitions rarely take into account the value placed by Indigenous youth on chosen or adopted kin as vital support networks. A response in many ways to the realities of ongoing colonization and intergenerational trauma that are experience by Indigenous youth in Canada.

Together, these concerns and critiques point to what participants regarded as the larger problem in PHIR more generally, which is that in most cases, it is neither youth-centred nor youth-determined. Community engagement is considered essential in PHIR, as it is critical to identifying health priorities and to ‘develop [ing] partnerships to enhance the success of a study’ ([32]:6). In practice however, Indigenous youth are not always granted this courtesy, and in their stead, others are engaged to set priorities and lead efforts. This reality highlights a power differential between researchers and community [32] that must be addressed by those interested in PHIR with Indigenous youth communities. In this respect, NYSHN provides a strength-based example of Indigenous youth leadership, in that they apply a decolonizing lens to all of their work, actively centering the concerns and worldviews of Indigenous youth and modeling youth leadership.

The participants in this study, similar to the Indigenous youth engaged in previous work [6, 29], are competent and able to define and recognize health and wellbeing, and its absence, both for themselves as well as their peers. Participants also shared nuanced understandings of the influences of colonialism and intergenerational trauma on their identities and experiences. For NYSHN, these critical understandings are integral to how they approach interventions related to Indigenous youth health and wellness, e.g. harm reduction. Going forward, non-youth (e.g. allies, accomplices and mentors) need to acknowledge these strengths and create opportunities for Indigenous youth to be empowered to self-determine. Non-youth can do so by creating and facilitating comprehensive, sustainable and accessible funding and culturally safe space for Indigenous youth to take on leadership roles in PHIR. Based on these findings, NYSHN has developed a handout that Indigenous youth can share with researchers and funders (See Additional file 3).

**Fig. 3:**
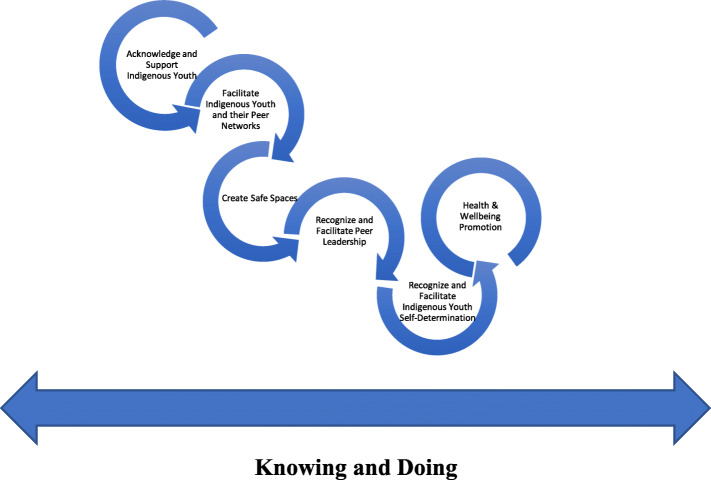
Knowing and doing: health and wellbeing of Indigenous youth

A key insight that emerged from this study was that participants did not differentiate between the stages of PHIR; public health research and health promotion activities were conceptualized one and the same. Knowing (research) and doing (service provision) were considered to be deeply interconnected and requiring the expertise and full involvement of Indigenous youth, as leaders and partners throughout. Knowing and doing with Indigenous youth, then, should not be approached as discrete activities. Colonialism has influenced the decontextualizing of knowledge from context and community, resulting in a schism between Indigenous knowing and doing [22]. Indigenous knowledge translation, or the bridge between knowing and doing, can be understood as the ‘Indigenously-led sharing of culturally relevant and useful health information and practices to improve Indigenous health status, policy, services, and programs’ ([33]:24–25). This definition aligns with the principles of Indigenous self-determination where ‘Indigenous leadership is essential’ [29]. Figure 3 illustrates how the stages of knowing and doing are a continuous process, while mapping how to initiate and sustain self-determined Indigenous youth leadership within the process. Crucial to this process is acknowledging that recognizing and fulfilling the initial stages of support, facilitation, and safe space, is necessary to achieve the IS and/or PHIR goals of health and wellbeing.

Finally, participants in this study also agreed that IS and PHIR need to be accountable to the Indigenous youth community. The individuals, agencies and institutions that are involved in directing and practicing research must be equipped to accept when they are told no, or to stop research when and if it is identified by youth as either harmful or lacking these goals, one possible accountability mechanism is for Indigenous youth organizations and allies to work in partnership and rate research, services and programs intended for Indigenous youth through audits. In a similar example, the Native Women Association of Canada has issued report cards for the Missing and Murdered Indigenous Women Inquiry [34]. Indigenous youth-based organizations such as NYSHN could audit research, services and programs on how well they respond to Indigenous youth and prioritize Indigenous youth’s expertise and leadership. Another potential mechanism would be to establish and fund Indigenous youth-led advisory committees that can participate in research priority setting exercises, research ethics reviews and evaluation/assessment of research and programs and services where Indigenous youth form the population of interest.

### Strengths and limitations

The work described here is conducted and governed in full partnership with NYHSN – where NYSHN members and Indigenous youth were directly involved in- and central to- all stages of the research project (i.e. conceptualization through to dissemination). This process is realized and governed though a community research agreement. As a result, youth-determined concepts and experiences are well-represented in the work. This approach is critical to all public health research and programming with Indigenous youth.

Data collection for this case study was limited to NYSHN leadership and NYSHN members and may be biased towards their experiences; the experiences of Indigenous youth more broadly, in different settings, are rich and diverse and will vary. As such, the research presented here can provide insight but is not necessarily generalizable. This limitation however, illustrates a larger concern which is the limited number of partnerships between Indigenous youth and/or their representative organizations with the larger public health research and services community.

## Conclusion

As emerging frameworks that prioritize context, end users, and community engagement, IS and PHIR are increasingly relied upon in the field of public health and amongst policy circles to improve population health. Similar to knowledge translation, implementation research more generally is viewed as a way to address the ‘know-do-gap’, forming a critical link between what should happen in theory and what actually happens in practice. In this respect, it attempts to address different aspects of implementation, including social and contextual factors [9, 32]. While these frameworks are certainly beneficial, they have been largely developed outside of Indigenous contexts and are thus subject to the same criticism put forth by Indigenous scholars [3, 22] about knowledge translation. This qualitative case study demonstrates and documents how Indigenous youth, as NYSHN, can and do practice their own form of PHIR in the realm of mental health promotion for 2SLGBTTIQQIA and Gender Non-Conforming Indigenous youth. This study is a critical reminder of the need to centre Indigenous youth throughout PHIR. Importantly, we highlight promising practices (Table 2, Supplemental Material) learnt in this case study, as those featured in NYSHN-led research such as the Sexy Health Carnival. These promising practices emphasize that researchers need to: recognize Indigenous youth as partners and leaders in research initiatives, meet youth where they are at; ensure and promote cultural safety, support Indigenous youth efforts to self-determine, ensure fair and adequate distribution of PHIR resources; compensate Indigenous youth as research partners; and, prioritize Indigenous youth-determined frameworks along with agreed upon accountability mechanisms.

## Supplementary Information

**Additional file 1.**

**Additional file 2.**

**Additional file 3.**

## Data Availability

The qualitative datasets generated and/or analyzed during the current study are not publicly available due to privacy and ethics restrictions but may be made available from the corresponding author billie.hardy@unityhealth.to with permissions from NYSHN on reasonable request.

## Abbreviations

2SLGBTQQIA: Two Spirit, lesbian, gay, bisexual, transgender, queer, questioning, intersex and allies
CIHR: Canadian Institutes for Health Research
FNIM: First Nations, Inuit, and Métis
IS: Implementation Science
NYSHN: Native Youth Sexual Health Network
PHIR: Public Health Implementation Research
