# Supplementary material for: Meeting Indigenous youth where they are at: knowing and doing with 2SLGBTTQQIA and gender non-conforming Indigenous youth: a qualitative case study
Source: BMC Public Health. 2020 Dec 7;20:1871. doi: 10.1186/s12889-020-09863-3 (PMC7720630; doi:10.1186/s12889-020-09863-3)
Supplement: Supplementary file 1 — Additional file 1. [file 12889_2020_9863_MOESM1_ESM.pdf]

<u>Research, Data, Statistics, and Publication Agreement</u>

Between

Native Youth Sexual Health Network ("NYSHN")

and

St. Michael's Hospital ('SMH")

and

Dr. Janet Smylie ("Principal Investigator")

Project: **Youth-Governed Approaches to Mental Health Promotion and Suicide Prevention for Two Spirit, LGBTTQQIA and Gender Non-Conforming Youth**

THIS AGREEMENT MADE IN DUPLICATE this May 29th, 2015 WITHNESSETH AS FOLLOWS

PURPOSE OF AGREEMENT:

The purpose of this agreement is to ensure that the Pathways Implementation Research Team (IRT) project**, "Youth-Governed Approaches to Mental Health Promotion and Suicide Prevention for Two Spirit, LGBTTQQIA and Gender Non-Conforming Youth"** is respectful to the cultures, languages, knowledge, values, and rights to self-determination of NYSHN. This agreement will also provide a framework for the use of data collected during the IRT project. This agreement supports principles of Indigenous collective and self-determined data management and governance. The agreement supports the information needs of NYSHN, the principal knowledge users as well as acknowledging the desire of Principal Investigator and her research team to conduct this collaborative research.

AGREEMENT PRINCIPALS:

- Maintain mutual respect and accountability between the parties;
- Recognize the complementary and distinct expertise, responsibilities, mandates, and accountability structures of each party;
- Ensure the highest standards of research and community ethics, including the acknowledgement of NYSHN specific principles of self-determined data management;
- Respect the individual and collective privacy rights of NYSHN personnel, including youth leaders, council members and network members;

- Recognize the value and potential of research that is scientifically and culturally validated and safe;
- Recognize the value of capacity building at all levels;
- Support NYSHN processes, including the analysis and dissemination of results;
- Support the involvement of NYSHN in project activities so that the results will be meaningful for the work of NYSHN youth, in accordance to the values and principles of NYSHN (see WHAT EW BELIVE IN on NYSHN website) http://www.nativeyouthsexualhealth.com/whatwebelievein.html)
- Work together to seek future funding and sustainable opportunities to continue this work and ensure longevity beyond project deadlines(s)

PROJECT DESCRIPTION (see Appendix A)

AGREEMENT:

AND WHEREAS NYSHN is developing a policy framework of principles for data collection, self-determined data management, analysis, and dissemination;

AND WHEREAS the NYSHN principles will be articulated in a written format;

AND WHEREAS NYSHN wishes to use this opportunity to build research capacity and/or provide research opportunities to its members and staff by working in collaboration with SMH;

AND WHEREAS NYSHN would like to maintain a positive and good faith relationship with SMH;

NOW THEREFORE SMH, NYSHN AND Principle Investigator covenant and agree as follows for the consideration of the sum of [Removed] NYSHN by SMH, and other valuable consideration, the receipt and sufficiency of which is hereby acknowledged;

1. SMH and Principal Investigator acknowledge that any and all data collected by NYSHN as a result of this research project is rightfully owned by NYSHN. Utilization of the data collected for the purpose and by the means outlined in the research proposal is acknowledged and granted by NYSHN to SMH and Principal Investigator in accordance with the terms and conditions contained in this agreement.

2. NYSHN agrees to undertake the research roles, responsibilities, and activities described in Appendix B with the support and mentorship of the Principal Investigator and Well Living House.

3. SMH and Principal Investigator agree to compensate NYSHN for these research roles, responsibilities and activities using the Canadian Institutes of Health Research ("CIHR") funds (CIHR budget incorporated herein as Appendix D) allocated for these undertakings described in Appendix E.

4. The Principal investigator will work in partnership with NYSHN on IRT project and they will jointly be acknowledged in any and all publications, reports, documents, or other written material from which this data is utilized. Representatives from SMH, Principal Investigator and NYSHN will be involved in the complete research process. SMH and Principal Investigator will provide NYSHN the opportunity for review of and feedback on any research reports before the submission of reports from publication. NYSHN will be provided four (4) weeks to review the research results and accompanying manuscript. In the event that NYSHN and SMH cannot agree on the content of the written report, NYSHN will be invited to write their own addendum to the report, which will be included as part of the overall report in all publications and/or pertinent published or produced materials.

5. NYSHN are the rightful owners of all data collected by NYSHN and/or from NYSHN staff or delegates in the course of this project. SMH and Principal Investigator will require NYSHN consent to maintain a copy of the collected data with Principal Investigator's databank. SMH and Principal investigator will be required to protect the data from unauthorized use and act as stewards on behalf of the rightful owner. NYSHN have provided prior consent to Principal Investigator of the Centre for Research on Inner City health at St. Michael's Hospital to maintain a copy of the collected data generated by this project in accordance with the Study protocol reviewed and approved by SMH research ethics board ("SMH REB") and NYSHN management for the purpose of publishing research reports as set out herein and having access to a copy of the source collected data of such research reports.

6. This agreement is in force from the date of the last authorizing signature below. Any party may terminate this agreement in the event of a material breach by any other party, or for any reason upon sixty (60) days written notice to the other parties.

7. The parties agree that this agreement is irrevocable and shall ensure to the benefit of any be binding upon the parties and their employees, administrators and legal and personal representatives.
8. The parties represent that they understand and agree to the terms contained within this agreement and such performance will not be unreasonably withheld.

9. The parties declare that they have been given the opportunity to obtain independent legal advice with respect to the details of the terms evidenced by this agreement and confirm that they are executing this agreement freely and voluntarily.

10. NYSHN, SMH and Principal Investigator agree to perform their respective study activities in accordance with the research proposal as approved by the SMH REB, and all applicable laws, regulations and guidelines, including without limitation, the 2nd edition of the Tri-Council Policy Statement, “Ethical Conduct for Research involving Humans” published by the CIHR, the Natural Sciences and Engineering Research Council of Canada and the Social Sciences and Humanities Research council of Canada dated December 2014, as amended from time to time, the CIHR Guidelines, “Guidelines for Health research involving Aboriginal People”, the What We Believe In[1] section of NYSHN website, and as amended from time to time.

11. The Parties confirm their respect for the privacy of individual participants in the research project. NYSHN, SMH and Principal investigator agree to follow applicable privacy laws including, but not limited to, *Personal Information Protection and Electronic Documents Act* (“PIPEDA”) and Ontario’s *Personal Health Information Protection Act*(“PHIPA”), and regulations and to notify each other if either receives a complaint about breach of privacy

12. Neither party shall use the name of the other party or its staff in any publication, news release, promotion, advertisement, or other public announcement, whether written or oral, that endorses services, organizations or products, without the prior written consent of the party whose name is to be used

13. The Parties confirm that if they transmit this agreement by facsimile or such device, that the reproduction of signatures by facsimile or such similar devices will be treated as binding as if originals and undertake to provide all parties with a copy of this agreement bearing original signatures forthwith by courier.

14. Notice to each party shall be sent to:

**SMH:**<br>St. Michael's Hospital<br>Attn: Dalton Charters<br>Director, Research Operation<br>Office of Research Administration<br>St. Michael’s Hospital<br>30 Bond St.<br>Toronto, Ontario		**NYSHN:**<br>Native Youth Sexual Health Network<br>Attn: [Name Removed<br>2345 Yonge St.<br>PO Box 26069 Broadway<br>Toronto, Ontario<br>Canada<br>M4P 0A8

[1] Native Youth Sexual Health Network. (2015).http://www.nativeyouthsexualhealth.com/whatwebelievein.html

M5B 1W8<br><br>**Principal Investigator:**<br><br>Dr. Janet Smylie<br>Centre for Research on Inner City<br>St. Michael's Hospital<br>30 Bond St.<br>Toronto, Ontario<br>M5B 1W8		

15. This agreement may be executed in counterpart. Copies collectively bearing the signatures of all parties shall constitute the fully executed agreement.

SIGNATURES: [Removed]

APPENDIX A: PROJECT DESCRITION

The proposed Pathways Implementation Research Team (IRT) project is focused on youth suicide prevention. Rates of suicide, self-harm and self-injury are increasing among Indigenous youth in Canada. Although there are enormous variations across communities, overall suicide rates are five to seven times higher for First Nations youth than for non-Aboriginal youth and 11 times the national average among Inuit youth. Rates of suicide cannot be broken down by sexual orientation or gender identity as coroner reports and death records do not require this information. However, elevated rates of related risk factors among Two-Spirit, gay, lesbian, bisexual, or transgendered Indigenous youth indicate higher levels of suicide risk as compared to heterosexual Indigenous youth. As highlighted in a growing body of literature, Two-spirit people are more likely to experience racism, violence and assault, which can result in loss of trust, safety and self-worth, increased fear and isolation as well as depression, anxiety, pain, poorer physical health and symptoms of post-traumatic stress.

The occurrence of suicide and self-harm among Two-Spirit and gender non-conforming Indigenous youth in Canada has received limited attention. This represents an alarming gap in knowledge given the disparate rates of suicide among the broader population of Indigenous youth in Canada. Moreover, mainstream mental health services and mental health promotion messaging around suicide, self-harm and self-injury do not reflect the experiences, knowledge or needs of Indigenous youth, and have not been effective in addressing the impacts of violence that Indigenous youth witness or experience on a daily basis. Importantly, mainstream mental health narratives fail to recognize and respect the right of Indigenous youth to self-determination.

The **overarching goal** of this Phase 1 IRT youth-directed research project is to identify population level interventions for suicide and self-injury prevention and mental health promotion that are relevant, effective and potentially adaptable for populations of Two Spirit, gender non-conforming and LGBTTQQIA[2] youth. The specific **objectives** of the proposed research include:

1. To build on and strengthen existing relationships and governance structures between Indigenous youth communities, NYSHN, the WLH and additional academic researchers with relevant expertise.

2. To provide a safe and healthy research environment for Indigenous youth through the development of a peer support training module on mental health promotion and suicide prevention.

3. (i) To engage with Indigenous youth to articulate guiding assumptions and recommendations regarding the re-framing of language addressing suicide, self-harm and self-injury among Two Spirit, LGBTQQII and gender non-conforming Indigenous youth. (ii) To actively and iteratively

[2] LGBTTQQIA: An acronym used to describe lesbian, gay, bisexual, transgender, transsexual queer, questioning, intersex and asexual self-identifying individuals.

disseminate these guiding assumptions and recommendations using traditional and innovative social media formats.

4. To conduct a systematic review of the published and grey literature on existing mental health promotion and suicide, self-harm and self-injury prevention services in Indigenous contexts.

5. To further investigate identified promising and best practices including peer support training and models of healing such as rites of passage and arts-based methodologies through community based key informant interviews and contextual documentation and analysis.

6. To assess the feasibility of scaling up select promising practices, including costing and a critical assessment of contextual factors, including specific barriers to and facilitators of health and wellness and the role of underlying determinants of health including colonization, poverty, social exclusion, family stress, and health literacy.

APPENDIX B: NYSHN RESEARCH ROLES, RESPONSIBILIES, AND ACTIVITIES

- Assist in co-development of this Research Agreement for the IRT Project
- Lead the co-organization and documentation of monthly teleconferences with the research team
- Co-develop an updated project work plan that details activities, timelines, and deliverables
- Provide a safe and healthy research environment for Indigenous youth to learn and meaningfully participate as well as lead and co-facilitate activities
- Provide direct mentorship to youth leaders through the duration of the project
- Develop a peer support training module on mental health promotion and suicide prevention
- Pilot this peer support training module at one or more of the four youth gatherings listed below
- Provide input and feedback on a systematic literature review of existing mental health promotion and suicide, self-harm and self-injury prevention services in Indigenous contexts
- Support the documentation of this literature review into an Indigenous youth friendly format as well as an academic journal submission.
- Co-ordinate two youth gatherings in Toronto with youth leaders, knowledge keepers and research project team. This includes organizing youth and non-WLH researcher travel, accommodation and meals and facilitation of all activities for both events.
- Arrange for an Indigenous knowledge keeper to present on rites of passage at the first Toronto gathering
- Co-create documents for the youth gatherings:
    1. First gathering: a youth survey geared towards understanding participant youth expectations of the research project

2. Second gathering: a sustainability plan for the scaling up of select promising practices that arise from the research project findings

3. Both gatherings: meeting reports

- Facilitate and document two discussion group conversations at two youth council meetings (Meeting dates and locations TBD). Discussion topic to be covered: guiding assumptions and recommendations regarding the re-framing of language addressing suicide, self-harm and self-injury among Two Spirit, LGBTQQII and gender non-conforming Indigenous youth

- Co-create recommendations on teachings related to suicide and self-harm, drawing on youth council discussions

- Co-create a press release and fact sheets regarding these recommendations on reframing suicide and self-harm.

- Lead engagement with social media platforms to disseminate these recommendations and raise awareness and dialogue on reframing suicide and self-harm within Indigenous youth populations

- Support youth to hold youth-led small group gatherings in their respective communities

- Co-organize webinar to review/present on up to 5 promising interventions on suicide and self-harm

- Co-conduct with Well Living House an in-depth analysis of up to 5 promising interventions. This includes NYSHN peer researcher arranging for in person and/or virtual site visit and co-conducting key informant interviews and environmental scan of local context and analysis and documentation of gathered information in the form of an online fact sheet and academic article.

- Co-develop and finalize a feasibility matrix regarding identified promising practices that includes costing and a critical analysis of key contextual factors that could impact implementation

- Co-develop and submit with WLH a CIHR research proposal application for a Pathways Implementation Research Project Component 2

- Facilitate 4 dedicated workshop sessions at the youth gatherings in Toronto and/or youth council meetings that focus on:

    1. Indigenous youth-determined conceptual models and practical strategies for academic-community data sharing and research partnerships and platforms;

    2. Indigenous youth- determined understandings of the barriers to and facilitators of health and wellness promoting behaviours, including the role of underlying determinants of health including racism, family and community relationship and health literacy and existing systems of health knowledge and practice

    3. Indigenous youth directed approaches to the assessment of promising practices

    4. Indigenous youth direct approaches to cross-community sharing of best practices

- Have a NYSHN researcher work with a WLH researcher to synthesize and document the findings of these workshops

- Work in partnership with Well Living House Research Team to develop and finalize a case study report that describes the implementation of this research project and documents Indigenous youth led health promoting research processes and activities (drawing on input from 4 gatherings)

Note: Final three activities/deliverables are also linked to the NYSHN-SMH CIHR Applied Public Health Chair activities/deliverables

APPENDIX C: SMH RESEARCH ROLES, RESPONSIBILITIES, AND ACTIVITIES

The SMH research team, under the direction and leadership of Dr. Janet Smylie will work in partnership with NYSHN and with the meaningful participation of NYSHN research team members to:

- Lead in development of this research agreement for IRT Project, as well as mentor and support NYSHN to carry this out

- Lead in the co-development of systematic literature review of existing mental health promotion and suicide, self-harm and self-injury prevention services in Indigenous contexts

- Lead in the documentation of this literature review into an Indigenous youth friendly format as well as an academic journal submission.

- Assist in organizing and documentation of monthly teleconferences with research team

- Assist in the co-ordination of two youth gatherings in Toronto with youth leaders, knowledge keepers and research project team.

- Support the creation of documents required for these meetings

- Attend two youth council meetings (dates and locations TBD) and assist as needed in the facilitation and documentation of discussions of guiding assumptions and recommendations regarding the re-framing of language addressing suicide, self-harm and self-injury among Two Spirit, LGBTQQII and gender non-conforming Indigenous youth

- Co-create recommendations on teachings related to suicide and self-harm, drawing on youth council discussions

- Co-create a press release and fact sheets regarding these recommendations on reframing suicide and self-harm.

- Support engagement with social media platforms to disseminate these recommendations and raise awareness and dialogue on reframing suicide and self-harm within Indigenous youth populations

- Co-organize webinar to review/present on up to 5 promising interventions on suicide and self-harm

- Co-conduct with NYSHN an in-depth analysis of up to 5 promising interventions. This includes co-conducting key informant interviews and environmental scan of local context and analysis and documentation of gathered information in the form of an online fact sheet and academic article.

- Co-develop and finalize a feasibility matrix regarding identified promising practices that includes costing and a critical analysis of key contextual factors that could impact implementation. Feasibility matrix to be developed based off of practices/factors identified by youth.

- Co-develop and submit with WLH a CIHR research proposal application for a Pathways Implementation Research Project Component 2

- Attend and support as needed the facilitation of 4 dedicated workshop sessions at the youth gatherings in Toronto and/or youth council meetings that focus on:

    1. Indigenous youth-determined conceptual models and practical strategies for academic-community data sharing and research partnerships and platforms;

    2. Indigenous youth- determined understandings of the barriers to and facilitators of health and wellness promoting behaviours including the role of underlying determinants of health including racism, family and community relationship and health literacy and existing systems of health knowledge and practice

    3. Indigenous youth directed approaches to the assessment of promising practices

    4. Indigenous youth direct approaches to cross-community sharing of best practices

- Work with NYSHN researcher/project manager to synthesize and document the findings of these workshops in a form that is meaningful for NYSHN and youth involved

- Work in partnership with NYSHN to develop and finalize a case study report (in a form that is identified as meaningful by NSHYN/youth) that describes the implementation of this research project and documents Indigenous youth led health promoting research processes and activities (drawing on input from 4 gatherings)

- Work with NYSHN to create a case study report that also is held within the larger process of their work and transition phase.

Note: Final three activities/deliverables are also linked to the NYSHN-SMH CIHR Applied Public Health Chair activities/deliverables
