# Supplementary material for: Meeting Indigenous youth where they are at: knowing and doing with 2SLGBTTQQIA and gender non-conforming Indigenous youth: a qualitative case study
Source: BMC Public Health. 2020 Dec 7;20:1871. doi: 10.1186/s12889-020-09863-3 (PMC7720630; doi:10.1186/s12889-020-09863-3)
Supplement: Supplementary file 2 — Additional file 2. [file 12889_2020_9863_MOESM2_ESM.pdf]

<u>Research, Data, Statistics, and Publication Agreement</u>
Between
Native Youth Sexual Health Network ("NYSHN")
and
Centre for Research on Inner City Health (CRICH) St. Michael's Hospital ("SMH")
and
Dr. Janet Smylie ("Principal Investigator")

Project: **Applied Public Health Chair in Indigenous Health Information and Knowledge Systems:Clearing the Pathways to Health Equity: Applied Public Health Chair in Indigenous Health Information and Knowledge Systems**

THIS AGREEMENT MADE IN DUPLICATE this day, March 9, 2015, WITNESSETH AS FOLLOWS:

PURPOSE OF AGREEMENT:
The purpose of this agreement is to ensure that the Applied Public Health Chair in Indigenous Health Information and Knowledge Systems program "**Clearing the Pathways to Health Equity: Applied Public Health Chair in Indigenous Health Information and Knowledge Systems**" ("Research Program") is respectful to the cultures, languages, knowledge, values, and rights to self-determination of NYSHN. This agreement will also provide a framework for the use of data collected during the Research Program. This agreement supports principles of Aboriginal collective and self-determined data management and governance. The agreement supports the information needs of NYSHN, as well as acknowledging the desire of Principal Investigator and her research team to conduct this collaborative research. It defines the opportunity(ies) to develop research capacity at NYSHN. NYSHN anticipates this research project will assist in program and service policy making, planning, delivery, and evaluation.

AGREEMENT PRINCIPALS:

- Maintain mutual respect and accountability between the parties;
- Recognize the complementary and distinct expertise, responsibilities, mandates, and accountability structures of each party;
- Ensure the highest standards of research ethics, including the acknowledgement of NYSHN specific principles of self-determined data management;
- Respect the individual and collective privacy rights of NYSHN personnel;
- Recognize the value and potential of research that is scientifically and culturally validated;
- Recognize the value of capacity building at all levels;
- Support NYSHN processes, including the analysis and dissemination of results.
- Support the involvement of NYSHN in the creation of any evaluation or tools so that the results will be meaningful for the work of NYSHN youth.

DRAFT

PROJECT DESCRIPTION (see Appendix A)

AGREEMENT:

AND WHEREAS NYSHN is developing a policy framework of principles for data collection, self-determined data management, analysis, and dissemination;

AND WHEREAS the NYSHN principles will be articulated in a written format;

AND WHEREAS NYSHN wishes to use this opportunity to build research capacity and/or provide research opportunities to its members and staff by working in collaboration with SMH;

AND WHEREAS NYSHN would like to maintain a positive and good faith relationship with SMH;

NOW THEREFORE SMH, NYSHN and Principal Investigator covenant and agree as follows for the consideration of the sum of [Removed] CDN dollars paid to NYSHN by SMH, and other valuable consideration, the receipt and sufficiency of which is hereby acknowledged;

1. SMH and Principal Investigator acknowledge that any and all data collected by NYSHN as a result of this research project is rightfully owned by NYSHN. Utilization of the data collected for the purpose and by the means outlined in the research proposal is acknowledged and granted by NYSHN to SMH and Principal Investigator in accordance with the terms and conditions contained in this agreement.
2. NYSHN agrees to undertake the research roles, responsibilities, and activities described in Appendix B.
3. SMH and Principal Investigator agree to compensate NYSHN for these research roles, responsibilities and activities using CIHR funds allocated for these undertakings described in Appendix D.
4. The Partners in Engagement and Knowledge Exchange (PEKE) grant holder from NYSHN will be [Removed] (PEKE Partners). Principal Investigator and the NYSHN PEKE Partners will work in partnership on the said project and that they will jointly be acknowledged in any and all publications, reports, documents, or other written material from which this data is utilized. Representatives from both SMH, Principal Investigator and NYSHN will be involved in the complete research process or to the level the representatives are directed to be involved.
5. SMH and Principal Investigator will provide NYSHN the opportunity for review of and feedback on any research reports before the submission of reports for publication. NYSHN will be provided four (4) weeks to review the research results and accompanying manuscript. In the event that the NYSHN and SMH

DRAFT

cannot agree on the content of the written report, NYSHN will be invited to write their own addendum to the report which will be included as part of the overall report in all publications and/or pertinent published or produced materials.

6. Utilizing the data gathered from this Research Program by SMH and Principal Investigator for secondary publishing will require specific written permission of NYSHN. SMH and Principal Investigator are to protect the data and act as stewards of this data on behalf of the rightful owner.
7. NYSHN are the rightful owners of all data collected. SMH and Principal Investigator will require NYSHN consent to maintain a copy of the collected data with Dr. Smylie's databank. SMH and Principal Investigator will be required to protect the data from unauthorized use and act as stewards on behalf of the rightful owner. NYSHN have provided prior consent to Principal Investigator of the Centre for Research on Inner City Health at St. Michael's Hospital to maintain a copy of the collected data generated by this project in accordance with the Study Protocol reviewed and approved by SMH research ethics board ("SMH REB") and NYSHN management for the purpose of publishing research reports as set out herein and having access to a copy of the source collected data of such research reports.
8. This agreement is in force from the date of the last authorizing signature below. Any party may terminate this agreement in the event of a material breach by any other party, or for any reason upon sixty (60) days written notice to the other parties.
9. The parties agree that this agreement is irrevocable and shall ensure to the benefit of and be binding upon the parties and their employees, administrators and legal and personal representatives.
10. The parties represent that they understand and agree to the terms contained within this agreement and such performance will not be unreasonably withheld.
11. The parties declare that they have been given the opportunity to obtain independent legal advice with respect to the details of the terms evidenced by this agreement and confirms that they are executing this agreement freely and voluntarily.
12. NYSHN, SMH and Principal Investigator agree to perform their respective study activities in accordance with the research proposal as approved by the SMH REB, and all applicable laws, regulations and guidelines, including without limitation, the 2nd edition of the Tri-Council Policy Statement, "Ethical Conduct for Research Involving Humans" published by the Canadian Institutes of Health Research, the Natural Sciences and Engineering Research Council of Canada and the Social Sciences and Humanities Research Council of Canada dated December 2014, as amended from time to time, and the Canadian Institutes of Health Research Guidelines, "Guidelines for Health Research Involving Aboriginal People", the [1]What We Believe In section of NYSHN website, all as amended from time to time

DRAFT

[1] Native Youth Sexual Health Network (2015). http://www.nativeyouthsexualhealth.com/whatwebelievein.html

13. The parties confirm their respect for the privacy of individual participants in the research project. NYSHN, SMH and Principal Investigator agree to follow applicable privacy laws including, but not limited to, *Personal Information Protection and Electronic Documents Act* ("PIPEDA") and Ontario's *Personal Health Information Protection Act* ("PHIPA"), and regulations and to notify each other if either receives a complaint about breach of privacy
14. Neither party shall use the name of the other party or its staff in any publication, news release, promotion, advertisement, or other public announcement, whether written or oral, that endorses services, organizations or products, without the prior written consent of the party whose name is to be used
15. The parties confirm that if they transmit this agreement by facsimile or such device, that the reproduction of signatures by facsimile or such similar device will be treated as binding as if originals and undertake to provide all parties with a copy of this agreement bearing original signatures forthwith by courier.
16. Notices to each party shall be sent to:

**SMH**:<br>St. Michael's Hospital<br>Attn: Dalton Charters<br>Director, Research Operations<br>Office of Research Administration<br>St. Michael's Hospital<br>30 Bond St.<br>Toronto, Ontario<br>M5B 1W8<br><br>**Principal Investigator:**<br><br>Dr. Janet Smylie<br>Centre for Research on Inner City<br>St. Michael's Hospital<br>30 Bond St.<br>Toronto, Ontario<br>M5B 1W8		**NYSHN**:<br>Native Youth Sexual Health Network<br>Attn: [Name Removed]<br>2345 Yonge St.<br>PO Box 26069 Broadway<br>Toronto, Ontario<br>Canada<br>M4P 0A8

17. This agreement may be executed in counterpart. Copies collectively bearing the signatures of all parties shall constitute the fully executed agreement.

SIGNATURES: [Removed]

DRAFT

APPENDIX A: PROJECT DESCRIPTION

The CIHR APH Chair research program “Clearing the Pathways to Equity” seeks to:

- Re-orient and further develop Indigenous population health interventions related to data sets, data sharing systems, and evaluation methods to enhance the implementation, assessment, and refinement of the interventions selected by the Pathways initiative and
- Apply best practices in Indigenous community based knowledge translation to augment the uptake and translation of the Pathways initiative.

The FIIRE-Forum for Indigenous Implementation Research and Evaluation is one of the two major Chair program activities. The FIIRE involves the development and implementation of a national Indigenous knowledge network to share and gather knowledge, further develop and articulate Indigenous approaches to population health intervention research and implementation sciences.

Outputs will include:

Four (4) policy papers about Indigenous governance and management of Indigenous health information; the critical importance of local Indigenous contexts and local ways of knowing and doing in Indigenous health research; best practices in Indigenous health service and program evaluation and best practices in sharing locally successful health interventions across Indigenous communities. The four papers will focus on each of the four states of PHIR/IS research:

a) Assessment and Surveillance

b) Understanding Causes and Context

c) Development and Evaluation

d) Scale-Up and Impact Evaluation

The FIIRE network will also be evaluated through conducting key informant interview and focus groups with network participants that will be conducted at baseline, mid- and endpoint, a method successfully used to evaluate cross-network knowledge exchange.

The second major Chair program activity is a series of case studies which will document Indigenous Implementation science/population health intervention research approaches and practice through a series of actual implementation research projects.

DRAFT

APPENDIX B: NYSHN RESEARCH ROLES, RESPONSIBILITIES, AND ACTIVITIES

FIIRE Network Activities

- Assist in co-developing developing draft MOU for the FIIRE
- Attend First FIIRE face to face meeting, to be orientated to FIIRE network and to finalize MOU
- Attend Quarterly FIIRE conference calls
- Attend annual face to face FIIRE Network Meetings including dialogue circles regarding relevant consensus document development activities
- Attend consensus document working group meetings
- Assist with feedback, input on systematic literature reviews to support consensus document development
- Participate in the evaluation of FIIRE Network; including baseline, midpoint & endpoint interviews and focus Groups (to be co-ordinated with annual face to face meetings)
- Attend biannual webinars
- Participate on a facilitated Listserv
- Assist with the analysis of FIIRE network evaluation data
- Consensus document development
- Assist with the documentation and evaluation of FIIRE network evaluation

Case Study Activities

- Work in partnership with Well Living House research team to document Indigenous youth led health promoting research processes and activities

DRAFT

APPENDIX C: SMH RESEARCH ROLES, RESPONSIBILITIES, AND ACTIVITIES

The SMH research team, under the direction and leadership of Dr. Janet Smylie will work in partnership with NYSHN and with the participation of NYSHN research team members to:

FIIRE Network Activities

- Assist in co-developing developing draft MOU for the FIIRE
- Coordinate the first FIIRE face to face meeting, prepare orientation to FIIRE network and facilitate the finalization of MOU
- Coordinate quarterly FIIRE conference calls
- Coordinate annual face to face FIIRE Network meetings including facilitating dialogue circles processes
- Coordinate consensus document working group meetings
- Coordinate and collect feedback, input on systematic literature reviews to support consensus document development
- Prepare draft consensus documents and other relevant background and meeting materials.
- Coordinate the evaluation of FIIRE Network; conducting baseline, midpoint & endpoint interviews and focus Groups (to be co-ordinated with annual face to face meetings)
- Coordinate biannual webinars
- Coordinate and manage and maintain a facilitated Listserv
- Coordinate the analysis of FIIRE network evaluation data
- Coordinate the documentation and evaluation of FIIRE network evaluation data

Case Study Activities

- Work in partnership with NYSHN and NYSHN youth research trainees/peer researchers to support the documentation of Indigenous youth led health promoting research processes and activities.

DRAFT
