# Supplementary material for: Meeting Indigenous youth where they are at: knowing and doing with 2SLGBTTQQIA and gender non-conforming Indigenous youth: a qualitative case study
Source: BMC Public Health. 2020 Dec 7;20:1871. doi: 10.1186/s12889-020-09863-3 (PMC7720630; doi:10.1186/s12889-020-09863-3)

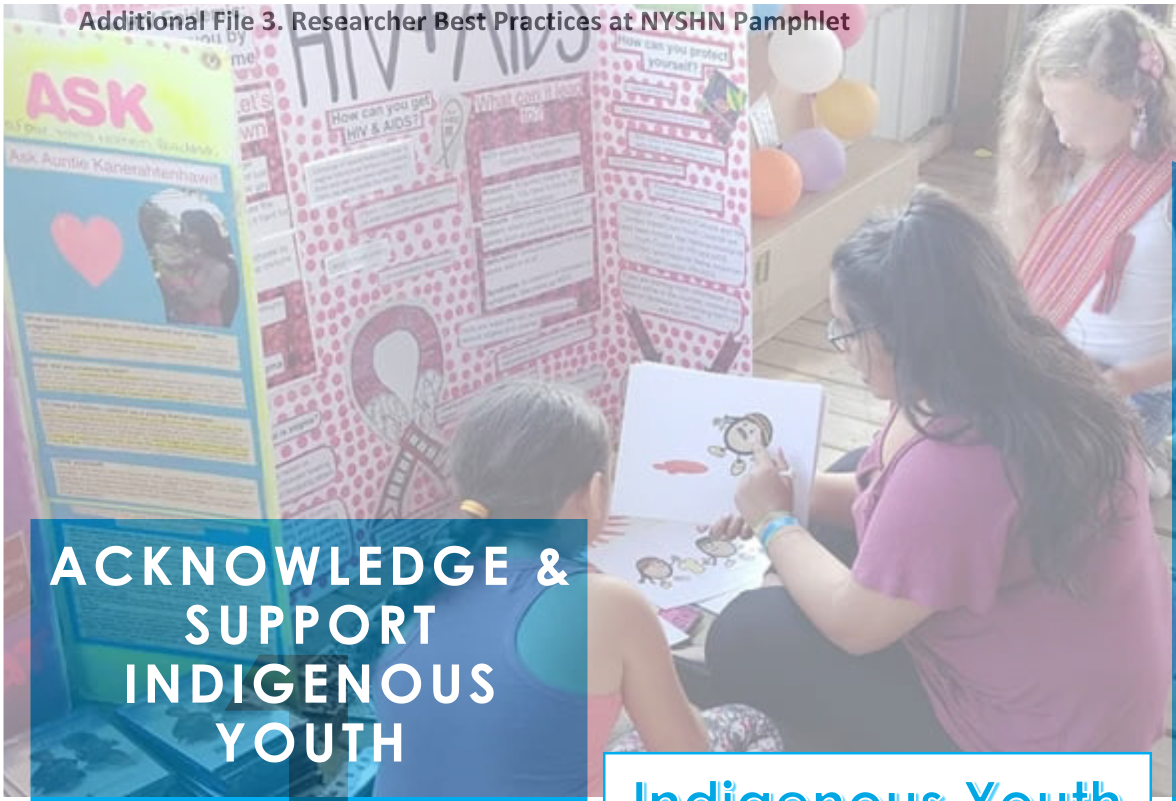

Indigenous Youth Want You To...

ACKNOWLEDGE & SUPPORT INDIGENOUS YOUTH

When youth feel supportred, they feel safe to be themselves, free from stigma & stereotypes forced upon us by colonial present society.

CREATE SAFE SPACES

Youth need the necessities, saftey, shelter, food. Most spaces for Indigenous youth are NOT safe, as they are predominantly caucasian with no representation on any level. When Indigenous youth gather there is an undenialble bond & trust. Safe spaces allow us to speak our truths & ask questions without stigma, jusgement, or racism.

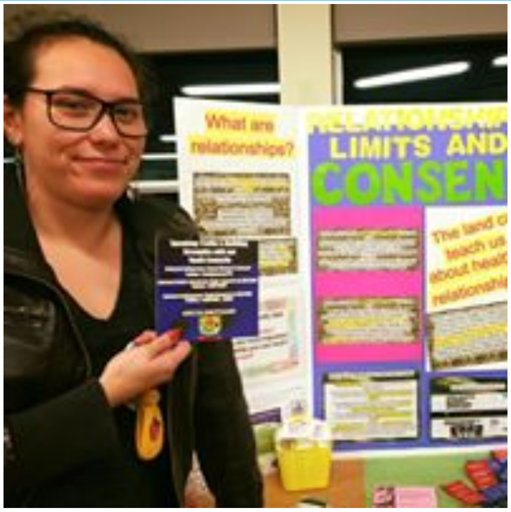

RECOGNIZE AND FACILITATE PEER LEADERSHIP

When Indigenous youth lead, other indigenous youth listen. We are not going to listen to some adult talk at us, we'd rather talk WITH other youth about our realities, as they are similar.

FACILITATE INDIGENOUS YOUTH & THEIR PEER NETWORKS

A great example in the two photos to the right. Youth using art in different ways, one creating patches with community; and a cultural meme!

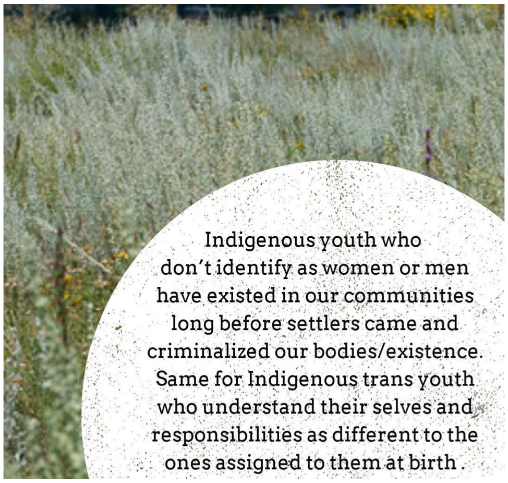

Indigenous youth who don't identify as women or men have existed in our communities long before settlers came and criminalized our bodies/existence. Same for Indigenous trans youth who understand their selves and responsibilities as different to the ones assigned to them at birth.

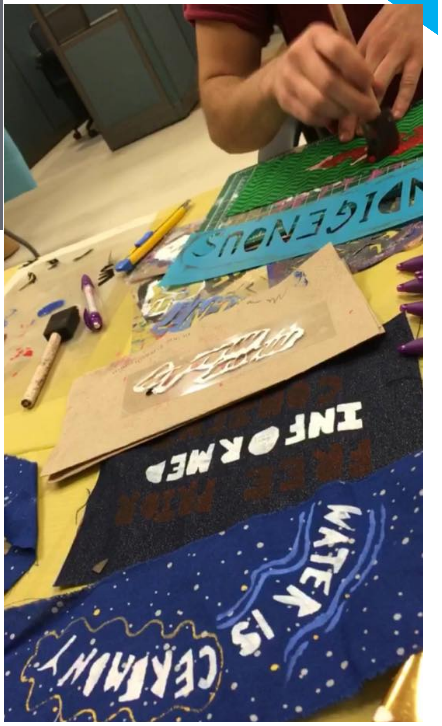

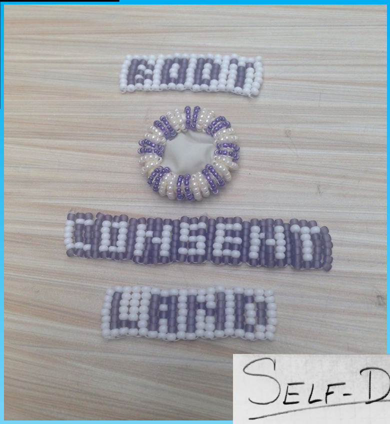

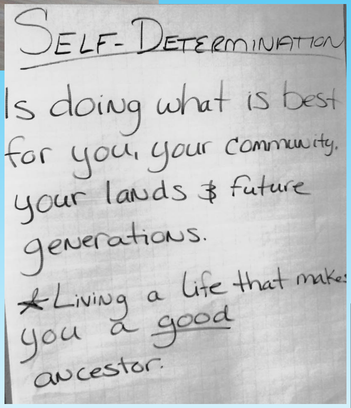

RECOGNIZE AND FACILITATE INDIGENOUS YOUTH SELF DETERMINATION

Indigenous youth have many gifts! It's hard and scary being yourself in this world filled with stigma, support Indigenous youth. They are the proffessionals in their own right. Wether it be their identity, safty, ideas, conversations, something they want to see happen in their community. Youth need to be supported and heard, not just listened to.

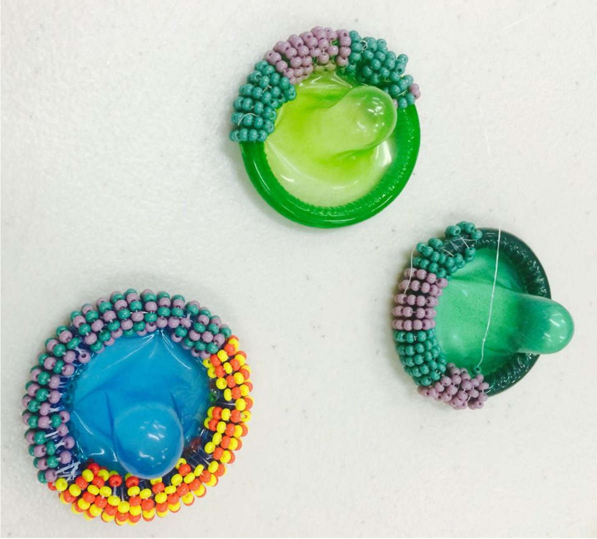

HEALTH & WELL BEING PROMOTION

The facts are as Indigenous youth, we do things differently! Normalizing talking about sex, condoms, STI's etc takes away the stigma attached to it. So by doing a cultural activity like beading condoms is a fun way to empower & rid stigma within our communities. **Each community is different, it's important to know the needs & cultural activities specific to them.**

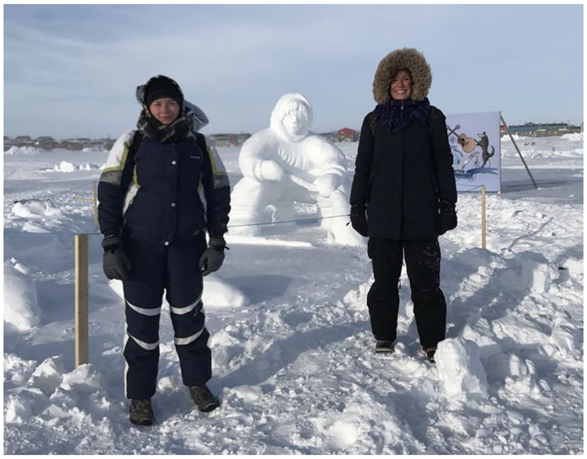

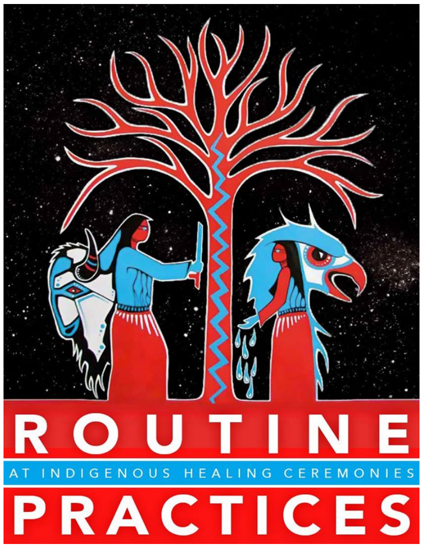

Supplement: Supplementary file 3 — Additional file 3. [file 12889_2020_9863_MOESM3_ESM.pdf]
